# The TOPGAME-study: effectiveness of extracorporeal shockwave therapy in jumping athletes with patellar tendinopathy. Design of a randomised controlled trial

**DOI:** 10.1186/1471-2474-11-28

**Published:** 2010-02-08

**Authors:** Johannes Zwerver, Evert Verhagen, Fred Hartgens, Inge van den Akker-Scheek, Ron L Diercks

**Affiliations:** 1 Center for Sports Medicine, University Center for Sports, Exercise and Health, University Medical Center Groningen, University of Groningen, Hanzeplein 1, 9700 RB Groningen, The Netherlands; 2 Department of Public and Occupational Health, The EMGO Institute for Health and Care Research, VU University Medical Center, Van der Boechorststraat 7, 1081 BT Amsterdam, The Netherlands; 3 Maastricht University Medical Center, Research school CAPHRI, Departments of Epidemiology and Surgery, PO Box 616, 6200 MD Maastricht, The Netherlands

## Abstract

**Background:**

Patellar tendinopathy is a major problem for many athletes, especially those involved in jumping activities. Despite its frequency and negative impact on athletic careers, no evidence-based guidelines for management of this overuse injury exist. Since functional outcomes of conservative and surgical treatments remain suboptimal, new diagnostic and therapeutic strategies have to be developed and evaluated.

Extracorporeal shockwave therapy (ESWT) appears to be a promising treatment in patients with chronic patellar tendinopathy. ESWT is most often applied after the known conservative treatments have failed. However, its effectiveness as primary therapy has not been studied in athletes who keep playing sports despite having patellar tendon pain.

The aim of this study is to determine the effectiveness of ESWT in athletes with patellar tendinopathy who are still in training and competition.

**Methods/design:**

The TOPGAME-study (Tendinopathy of Patella Groningen Amsterdam Maastricht ESWT) is a multicentre two-armed randomised controlled trial with blinded participants and outcome assessors, in which the effectiveness of patient-guided focussed ESWT treatment (compared to placebo ESWT) on pain reduction and recovery of function in athletes with patellar tendinopathy will be investigated. Participants are volleyball, handball and basketball players with symptoms of patellar tendinopathy for a minimum of 3 to a maximum duration of 12 months who are still able to train and compete. The intervention group receives three patient-guided focussed medium-energy density ESWT treatments without local anaesthesia at a weekly interval in the first half of the competition. The control group receives placebo treatment. The follow-up measurements take place 1, 12 and 22 weeks after the final ESWT or placebo treatment, when athletes are still in competition. Primary outcome measure is the VISA-P (Victorian Institute of Sport Assessment - patella) score. Data with regard to pain during function tests (jump tests and single-leg decline squat) and ultrasound characteristics are also collected. During the follow-up period participants also register pain, symptoms, sports participation, side effects of treatment and additional medical consumption in an internet-based diary.

**Discussion:**

The TOPGAME-study is the first RCT to study the effectiveness of patient-guided ESWT in athletes with patellar tendinopathy who are still in training and competition.

**Trial registration:**

Trial registration number NTR1408.

## Background

Patellar tendinopathy ('jumper's knee') is a clinical condition of gradually progressive activity-related pain at the insertion of the patellar tendon at the apex patellae [1]. Prolonged repetitive stress of the knee-extensor apparatus can lead to this common overuse tendinopathy in athletes from different sports [2]. The overall prevalence of patellar tendinopathy among elite and non-elite athletes is high and varies between 3 and 45% [3,4]. In sports characterised by high demands on speed and power for the leg extensors, such as volleyball and basketball a prevalence of respectively 44.6% and 31.9% has been reported [4]. In sports medicine centres patellar tendinopathy is one of the leading causes for athletes to consult physicians or physical therapists. Patellar tendinopathy often contributes to the decision to quit an athletic career and also causes mild but long-lasting symptoms after an athletic career [5]. The high prevalence, impact on sports performance, and chronic nature of the condition all mean that in some jumping sports, patellar tendinopathy may cause at least as much impairment in athletic performance as acute knee injuries [4].

There is no consensus on what is the most appropriate treatment for patellar tendinopathy [6,7]. Several conservative treatment modalities (e.g. physical therapy, anti-inflammatory medication, rest, exercise) and different surgical procedures for treatment of patellar tendinopathy have been described [8,9,6,7]. Overall, they have not been proven to be highly successful in relieving symptoms to such a degree that athletes can continue to participate in their sport at their full potential [8]. New treatment modalities for patellar tendinopathy have recently been introduced, based on the finding that the pathology underlying chronic (patellar) tendinopathies is not inflammatory tendinitis but a degenerative tendinosis due to a failed healing response [10].

In the last few years, extracorporeal shockwave therapy (ESWT) has also been used for the treatment of patellar tendinopathy. It seems to be a safe and promising treatment for patellar tendinopathy [11]. In most of the research on ESWT treatment for patellar tendinopathy to date, patients have been recruited in a referral-based specialist care setting. Moreover, ESWT is often only applied when other treatments have already failed. This means that most patients included in these studies have serious, chronic problems, generally to the extent that they had to stop sports participation entirely, or at least reduce their level of sports participation significantly. One can presume that patients in this stage have a decreased healing tendency and are possibly less responsive (more resistant) to all treatment modalities. To our knowledge, the effectiveness of ESWT has not been systematically investigated in athletes who have early symptomatic patellar tendinopathy and are still actively competing.

The aim of the TOPGAME study is to determine the effectiveness of ESWT on pain, symptoms and function, in athletes with patellar tendinopathy at an early stage of the disease who are still in training and competition.

## Methods/Design

### Design

**Figure 1:**
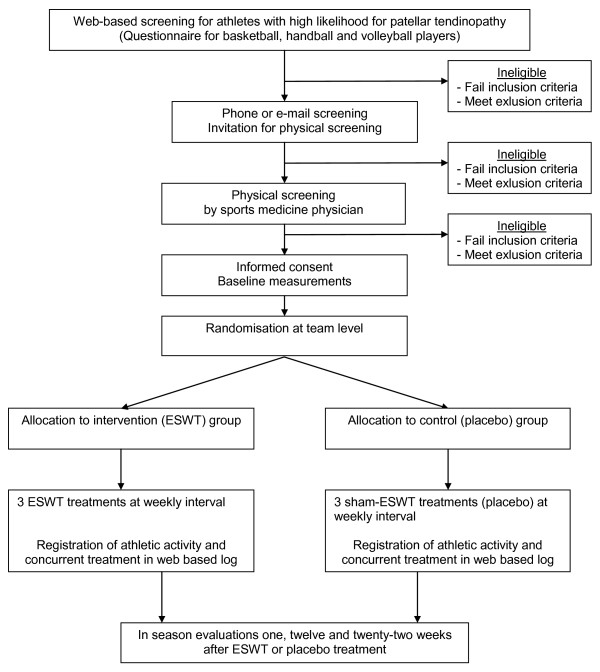
**TOPGAME Trial profile**. The Trial profile of the TOPGAME study.

The TOPGAME study (Tendinopathy Of Patella Groningen Amsterdam Maastricht ESWT) is a multicentre randomised controlled trial with blinded participants and outcome assessors, using a two-group repeated measures design with a treatment period of 2 weeks and a 22 week follow-up. Participants are randomized into an intervention ESWT group or a placebo control group. Recruitment of participants for the TOPGAME trial takes place in May-November 2008 and data collection starts in September 2008 (first half of the competition season). The ESWT and placebo treatments start in October 2008. After three ESWT or placebo treatments, the follow-up measurements take place at 1, 12 and 22 weeks after the final treatment, when athletes are still in competition. The trial profile is shown in Figure 1.

The study design, procedures and informed consent procedure were approved by the Medical Ethics Committee (Number 2008/052) of the University Medical Center Groningen (UMCG), the Netherlands. All participants have to provide written informed consent.

### Study population

Recruitment of the participants is facilitated by the Dutch Basketball Association (Nederlandse Basketbalbond, NBB), Dutch Handball Association (Nederlands Handbalverbond, NHV) and the Dutch Volleyball Association (Nederlandse Volleybalbond, NEVOBO). Through both an advertisement on their website and an e-mail sent to all their athletes (aged 18-35) and the coaches of the club teams, attention is drawn to the TOPGAME-study. Recruitment is further assisted by advertisements in local, regional and national newspapers and by advertising at tournaments and games.

All athletes are invited to fill out a questionnaire on sports participation and knee problems (including a pain map) on a specially designed website http://www.topgamestudie.nl. They are also asked for their willingness to participate in the study. After this questionnaire-based screening, participants with a high likelihood of having patellar tendinopathy are contacted by mail or phone and sent written information about the study. If they still want to participate, they receive an invitation for a consultation by one of the two sports medicine physicians of the study (FH, JZ) at a sports medicine practice in their neighbourhood. The sports medicine physicians examine all the participants and clinically establish the diagnosis of patellar tendinopathy using the criteria described below. Eligible participants are included in the study after informed consent. Thereafter, baseline measurements are carried out and athletes will be randomised to either the intervention (ESWT) group or the control (placebo) group.

### Inclusion and exclusion criteria

Male and female basketball, handball and volleyball players with the following criteria are eligible for inclusion:

1. History of knee pain in the patellar tendon or its patellar or tibial insertion (pointed out in an anatomical drawing of the knee) in connection with training and competition.

2. Symptoms for over three months in the actual season or in the second half of the previous season (January-May 2008) (to exclude acute inflammatory tendon problems and de novo partial ruptures).

3. Age 18-35 years (to reduce chances of osteochondrotic diseases like Sinding-Larsen-Johanson, Osgood-Schlatter and osteoarthrosis).

4. Palpation tenderness at the corresponding painful area.

5. VISA-P score < 80. The VISA-P (Victorian Institute of Sport Assessment - patella) score is a short questionnaire measuring the severity of patellar tendinopathy by assessing pain, function and ability to play sports [12,13].

Athletes are excluded if they suffer from acute knee or acute patellar tendon injuries, have chronic joint diseases or have signs or symptoms of other (co-)existing knee pathologies. Athletes who use drugs with a putative effect on patellar tendinopathy in the last year on daily basis (e.g. non-steroid anti-inflammatory drugs, fluorquinolones) or use anticoagulants, and athletes who had knee surgery or injection therapy with corticosteroids in the last preceding three months are also excluded. Athletes with contraindications for ESWT treatment (pregnancy, malignancy, coagulopathy) are also excluded from the study.

### Randomization

Volleyball, handball and basketball players with patellar tendinopathy are allocated randomly and blinded to an intervention group (patient-guided ESWT) or a control group (placebo treatment) by an independent statistician (EV) who is blinded for any baseline characteristics of the participants. Randomisation is performed before the first treatment by means of a computer-generated randomisation list (SPSS 16, Chicago, USA). The randomisation procedure takes place at team level, resulting in players from the same teams being allocated to the same group. While the different treatment methods within the study groups potentially have various immediately noticeable effects on the subjects (e.g. level of pain), this method of randomisation is chosen to keep the treatment group blinded to the subjects, and to avoid spill-over of the intervention. The independent physical therapists who administer the ESWT or placebo treatment are informed by the statistician about the group allocation. Group allocation is concealed from the athletes and the outcome assessor at all times during the trial.

### Intervention

ESWT treatment and placebo treatments will be given by five independent physical therapists at four different locations across the Netherlands.

#### ESWT treatment

The physical therapist will explain the treatment procedure to the athlete and will palpate the patellar tendon to find the most painful spot. ESWT is applied according to the guidelines of the International Society for Musculoskeletal Shockwave Therapy (ISMST) using a piezo-electric ESWT device (Piezowave, Wolf GmbH, Knittlingen, Germany). This will be administered in three sessions at one-week intervals using 2000 impulses at a frequency of 4 Hz. The energy flux density will be titrated according to individual pain tolerance up to a possible maximum of 0.58 mJ/mm2 (level 20). Treatment will start at level 5 (0.1 mJ/mm2). The athlete will be told that treatment can be painful but that there is inter-individual variability in pain perception. After every 100 impulses the physical therapist will ask the athlete, if he/she can tolerate the treatment. If he/she can, the physical therapist will increase the energy flux density by one level, up to the aforementioned maximum level. A transmission gel will be applied between the applicator and the focussing pad as well as between the focussing pad and the skin of the patient to optimise shockwave transmission to the patient. No local anaesthesia will be used, since Rompe and colleagues demonstrated that repetitive application of shockwaves is more effective without than with local anaesthesia [14]. The athlete will be in supine position with an extended knee and shockwaves will be focused on the painful zone in the tendon or insertion. The inferior pole of the patella will be tilted to focus on the dorsal insertion of the patellar tendon as well. The aforementioned treatment protocol was chosen based on our previous experience with ESWT application in patients with patellar tendinopathy (submitted). All patients in the pilot study tolerated this procedure well without adverse complications.

#### Placebo treatment

The treatment procedure for the athletes in the control group is nearly the same. Placebo treatment will be administered using the same device. The transmission gel will be applied between the focussing pad and skin of the patient, but not between applicator and focussing pad. This is invisible for the athletes, since it is inside of the device. In this way shockwaves are not or hardly conducted. Measurements of energy density provided by the manufacturer for this placebo set-up revealed negligible or only very low energy densities of less than 0,03 mJ/mm2 (Wolf GmbH, Knittlingen, Germany). The athlete will also be told that treatment can be painful but that there is inter-individual variability in pain perception. By pressing the applicator with focussing pad to the painful spot, athletes will also experience some pain. The physical therapist will also ask after every 100 impulses if the athlete can tolerate the ESWT treatment, but energy flux density will not increase during the treatment. Athletes in the placebo group will also hear the repetitive impulses generated by the ESWT-device, yet will be unaware of the dosage administered.

#### Concurrent sports participation and medical treatment

No restrictions will be given for both groups with regard to sport participation or concurrent medical treatment. If the athlete will experience an increase in pain in the first 48 hours after treatment he/she will be advised to take paracetamol up to a maximum dose of 3 dd 1000 mg for pain relief.

### Measurements

#### Baseline

After informed consent the following baseline measurements will be carried out:

#### Baseline questionnaire

The baseline questionnaire consists of three parts. Part 1 covers demographic variables such as name, address, age, gender, and e-mail address. Sports participation will be assessed in part 2 by using questions concerning type and level of sport and mean hours of sports participation and sport history. Information about medical history, knee injuries and previous medical treatment will be collected in the third part of the questionnaire.

#### VISA-P questionnaire

The primary outcome of the TOPGAME study is the self-reported VISA-P score [12]. The VISA-P score is a simple, reliable instrument for measuring the severity of patellar tendinopathy and is sensitive to small changes in symptoms. It was specifically designed for patellar tendinopathy, rating pain, symptoms, simple test of function and the ability to play sports. Six of the eight questions are scored on a scale from 0 to 10 points, with 10 representing optimal health. The maximum VISA score for an asymptomatic athlete is 100 points. Validity and reliability of the Dutch translation of the VISA-P score have been demonstrated recently [13].

#### VAS pain

Secondary outcome parameters are ratings of pain on a Visual Analogue Scale (VAS) during activities of daily living (ADL) and sports, and during functional tests like maximal jumping tests, triple hop test and the single leg decline squat (SLDS); the latter test, in which the athlete performs a single-leg squat to 60° of knee flexion on a 25° decline board ten times, was designed to preferentially load the patellar tendon [15,16].

#### Ultrasound

Greyscale ultrasound and Power/Colour Doppler characteristics of the patellar tendon (hypo-echogenity, diameter, calcifications, and degree of neovascularisation) will be collected by an experienced radiologist.

### Follow-up

Follow-up measurements for VISA-P and the VAS pain assessments will be carried out at 1, 12 and 22 weeks after the final treatment, when athletes are still in competition. Side effects and adverse reactions/events and the rate of overall treatment satisfaction will also be recorded. At 22 weeks after final treatment, ultrasound characteristics will be collected by the same experienced radiologist who is blinded to the athletes' group allocation. Further both groups of athletes will be recording their athletic activities and concurrent medical treatment on a weekly basis, using a web-based diary.

### Sample size

Sample size is calculated based on the VISA-P score. From previous investigations a baseline score of 64 points is expected in symptomatic athletes (95 points in athletes without patellar tendinopathy), with an SD of 19 points (2). (To our knowledge no data are available that describe the SD of the difference between the baseline and 6 month VISA-score.)

A 15-point difference in the VISA score between the treatment and placebo is considered to be clinically relevant. To detect a difference of 15 points on the VISA scale with an SD of 19, a power of 90% and an alpha of 5%, 34 subjects per group are needed.

The proposed treatment protocol without local anaesthesia and with patient-guided dosage (for pain tolerance) is chosen based on our previous experience with ESWT application in patients with patellar tendinopathy (J. Zwerver, submitted). All patients tolerated this procedure well without adverse complications; therefore we do not expect a higher-than-normal drop-out rate of athletes due to pain during the treatment. Assuming a drop-out rate of about 20% this would mean that a total of 86 subjects are required at baseline (43 in each group).

### Statistical analyses

Descriptive statistics (means and standard deviations, numbers and percentages) will be used to describe the characteristics of the intervention and control group and the outcome variables at the three measurement points. To evaluate potential group differences at the start of the study, baseline values will be analysed for differences between intervention group and control group. The effect of the ESWT treatment will be assessed using multilevel analysis. The multilevel analysis will be used to determine whether there is a difference on the primary and secondary outcome variables between the two groups over time. This statistical technique takes into account the dependency of observations of different subjects. Analyses will be adjusted for gender, age, type of sport, and baseline values of any other post hoc confounders. Analyses will be performed following the 'intention to treat' principle. Differences will be considered statistically significant at p < 0.05. All analyses will be done using SPSS version 16 (SPSS, Chicago, USA).

## Discussion

Despite its frequency and impact on athletic careers, and decades of research notwithstanding, management of patellar tendinopathy remains frustrating and unpredictable for both athletes and clinicians. ESWT appears to be a promising treatment method in patients with chronic patellar tendinopathy [11]. Up to now, only studies have been published in which ESWT has been used for the treatment of recalcitrant patellar tendinopathy in athletes who had several conservative treatments before and were finally referred to a sports medicine department for ESWT [11]. However, the effectiveness of ESWT has not been studied in the large group of athletes who continue sports participation with an early or mild symptomatic patellar tendinopathy. The TOPGAME study is the first RCT to study the effectiveness of patient-guided piezo-electrically generated ESWT in athletes with patellar tendinopathy who are still in training and competition. To our knowledge, it is also the first study that takes into account the training and competition load of the athletes using a web-based log.

This study will contribute to a better understanding of the effectiveness of ESWT as treatment for athletes with patellar tendinopathy who are still able to train and compete. By treating them at an early phase, we can get relevant information on whether it is possible to reverse or stop the progression of patellar tendinopathy, thereby preventing chronic impairment of athletic performance, work and daily activities.

## Conclusions

The TOPGAME study is the first RCT to evaluate the effectiveness of patient-guided ESWT in athletes with early-phase symptomatic patellar tendinopathy who are still in training and competition.

## List of abbreviations used

**TOPGAME**: Tendinopathy of Patella Groningen Amsterdam Maastricht ESWT; **ESWT**: Extracorporeal Shockwave Therapy; **RCT**: Randomised Controlled Trial; **NBB**: Nederlandse Basketbalbond (Dutch Basketball Association); **NHV**: Nederlands Handbalverbond (Dutch Handball Association); **NEVOBO**: Nederlandse Volleybalbond (Dutch Volleyball Association); **VISA-P**: Victorian Institute of Sport Assessment - patella.

## Competing interests

The authors declare that they do not have competing interest. Wolf GmbH (Knittlingen, Germany) supported the TOPGAME study by providing the ESWT devices. Neither the study nor any of the authors receives or received any funding or financial compensation from Wolf. Wolf has not been involved in the design of the study, nor will it be involved in analysis of the data and publications.

## Authors' contributions

JZ conceived of the idea, obtained funding for the study, developed the intervention and wrote the article. JZ and FH developed the design of this trial, recruited participants and are responsible for data acquisition. EV and IA provided advice on the study design and contributed to the content of the article. RD, EV and FH are co- applicants of the grant. FH and RD contributed to the content of the article. All authors read and approved the final manuscript.

## Pre-publication history

The pre-publication history for this paper can be accessed here:

http://www.biomedcentral.com/1471-2474/11/28/prepub
